# Community-engaged research as a pathway to oral health equity: insights from a Texas initiative

**DOI:** 10.3389/fpubh.2025.1548544

**Published:** 2025-03-18

**Authors:** Cody Price, Blair Williams, Andrea Mayo Jacks, Ankit Sanghavi

**Affiliations:** Texas Health Institute, Austin, TX, United States

**Keywords:** community, oral health equity, human-centered design, community-engaged research, system transformation

## Abstract

Solutions to advance oral health equity require a deeper understanding only achieved though partnership with the communities deeply impacted by barriers to care. While numerous studies and dental public health reports published over the years demonstrate a need for oral health equity, there is a paucity of literature regarding community engagement as a pathway to advancing oral health equity. As a human-centered design approach, Community-Engaged Research (CER) provides opportunities to engage communities as research partners, while developing trust and capacity for sustainable collaboration and participatory systems thinking. Building on literature and our experiences from leading a community-engaged oral health equity project in Texas, this perspective article offers actionable concepts of *trust, time*, and *co-design* to encourage the use of community-engaged practices that assess and address complex factors that impact oral health.

## Introduction

Numerous studies and dental public health reports published over the years demonstrate the importance of oral health due to the intrinsic connection to overall wellbeing, yet there is a paucity of literature regarding community-engaged research as a pathway to understanding and addressing barriers to oral and overall health (1). As a human-centered approach, Community-Engaged Research (CER) provides opportunities to engage communities as research partners (2–4), while simultaneously building trust (5), pathways, and capacity for sustainable solutions (6). Additionally, CER goes beyond the typical methods utilized for community health needs assessments or research by exploring research questions in authentic collaboration with the communities who experience the direct impacts of a given health concern. Put simply, where traditional research may be inadvertently exploitive of populations *as subjects of academic inquiry*, CER creates an opportunity to explore the issues *with the community as co-investigators*. Furthermore, evidence suggests the use of CER for health education, public health, social science (6), medicine (7), and oral health (2) research has benefited community (8), workforce development (9), and practice (10). Additionally, evidence and guidance from the American Dental Association (11, 12), Association of State and Territorial Dental Directors (13), United States Surgeon General (14), and Centers for Medicare and Medicaid (CMS) (15) support the use of community-based approaches. Despite the need, there is limited literature available on the practical application and benefits of CER in oral health (2, 16) to (re)build trust and capacity building for systems change (17–19). As such, this perspective article highlights our experiences with community-engaged research to offer practical solutions for those seeking to use CER as a pathway to advance oral health equity across the United States.

## Trust, time, co-design as essential components of community-engaged research

In 2023, we began an oral health initiative that sought: (1) a deeper understanding of the systems and conditions impacting oral health in Texas, and (2) action-oriented strategies to inform others working to advance population oral health in Texas and across the United States (20). The population and political contexts of Texas provide a unique opportunity to explore oral health needs by race, ethnicity, language, rurality, sexual orientation, gender identity, and disability status in a context where the voices of those inhabiting these identities have been historically marginalized in the oral health, public health, and political context. To accomplish this purpose and in recognition of the importance of CER to do so, we partnered with eight community-based organizations located throughout the state. As research partners on this initiative, the CBOs represented and advocated for their respective communities deeply impacted by barriers to oral healthcare. Together, we co-designed and conducted culturally and linguistically appropriate focus groups, contextualized findings during a sensemaking session to validate takeaways and limitations, and discussed strategies for next steps to include dissemination of the findings. In addition to this collaborative approach with CBOs, we also conducted key informant interviews with individuals leading oral health initiatives across the state. Insights from these interviews provided systems and political context. We triangulated findings from publicly available quantitative data, focus groups, and key informant interviews to develop actionable insights. While not exhaustive, the synthesis of community voices, context, and evidence informed the next phase of our oral health initiative. Drawing from our collective experiences with CER, this article focuses on three essential concepts to inform the practice of community-engaged research: (1) *trust*, (2) *time*, and (3) *co-design* (see also Table 1).

**Table 1:** Trust, time, and co-design: key concepts and practical examples of community-engaged research (CER).

**Concept**	**Description**	**Practical examples**
**Trust**	Genuine community engagement and collaboration are essential for building trust between the researcher and community. Trust results in a deeper understanding of complex systems and solutions to improve conditions for health and wellbeing.	• Engage community-based organizations (CBOs) to be research partners to ensure community is seen, heard, and valued throughout the research process. • Partner with community to create a safe space for participants.
**Time**	Building on the concept of trust, the concept of time includes honoring community participation with equitable compensation and appropriate project timelines to ensure collaboration between community and researcher at each stage (i.e., conceptualization, design, data collection, sensemaking, and dissemination).	• Provide equitable compensation for participation of research partners and community participants. • Consult with community research partners when developing project timelines to ensure adequate time for outreach and trust-building.
**Co-design**	Instead of traditional approaches that may be perceived as exploitive or prescriptive to communities that have been historically underrepresented in research, collaborative co-design approaches empower communities to safely explore root causes, solutions, and share visions of the future with researchers.	• Engage community research partners in decision-making across all stages of the research process. • Prepare for a robust sensemaking session with community research partners to contextualize takeaways and study limitations.

### Trust

As a human-centered approach, CER creates pathways for essential trust-building and a deeper understanding of the systems and conditions impacting oral health (5) that might not be achieved with traditional approaches (e.g., community health needs assessment or research project entirely led by an academic or research organization). Additionally, traditional research methods may be perceived by the study population as exploitive, transactional, or traumatic—particularly when they do not feel seen, heard, or valued throughout the research process. Without the valuable input and expertise from the impacted communities, methodologies that are entirely researcher-led may miss the opportunity to build trust that is crucial to understanding complex issues and developing sustainable solutions to address identified needs. As an alternative approach, the use of CER provides opportunities to engage the community in all phases of research—from design to dissemination, ensuring community voices are seen, heard, and valued. This collaboration between researchers and the community is essential to advance health equity, power scientific research, and implement effective solutions (5).

From our experiences with trust-building during this oral health initiative, we suggest researchers engage community-based organizations (CBOs) to serve as research partners given their roles as trust and cultural brokers within their respective communities and geographies. We selected and engaged our CBO research partners because they had well-established relationships and shared lived experiences with each of their respective populations—Texas-Mexico border communities (21, 22), rural residents of East Texas, members of historically Black neighborhoods of Houston and Austin (23, 24), and advocacy groups (25) representing faith leaders, Texans who have disabilities or special health care needs, and those who identify as lesbian, gay, bisexual, transgender, queer, or questioning, and other identities of the LGBTQ+ community. These organizations are well-positioned to speak to the unique needs of their communities, while collectively painting a portrait of the state as a whole.

In our partnership with CBOs, we also learned the importance of creating a safe space for participants. For example, we found focus group participants were more forthcoming in their responses due to their well-established relationships and shared experiences with the CBO. The CBOs facilitated conversations during the focus groups ultimately led to a deeper understanding of systems and conditions. Additionally, community members may be hesitant to participate in focus groups led by outside organizations due to fear or negative experiences. In an effort to mitigate this hesitancy, we co-designed transparent, plain-language communication strategies outlining the purpose of our research, consent process, and how we planned to utilize the findings while protecting their privacy. We feel this was essential to the success of our project and underscores the importance of trust and safety during recruitment, data collection, and reporting stages of research in ways that go beyond Institutional Review Board (IRB) requirements.

### Time

Building on the concept of *trust*, the concept of *time* is largely focused on the importance of the examples of equitable compensation and appropriate project timelines. In typical research approaches, participants may receive pre-determined incentives or compensation for attending a focus group. A project timeline might also be developed by the researcher without input from the community research partner—who may advise more time allocated to trust-building outreach. From our experience with CER on this project, we recommend that the compensation strategies and project timelines be developed with the CBO research partners.

Compensation strategies should include payment for the participating CBOs for their time as research partners, as well as the appropriate amount (and method) for community participants. In these compensation strategies, consider the valuable time participants take out of their busy schedules, transportation, food, and childcare. Also consider the logistics of compensation strategies to include the method of compensation (e.g., physical gift card vs. digital gift card, other forms of compensation). Consider also the importance of trust when discussing the logistics of compensation delivery, as participants may be hesitant to create digital footprints, or they may perceive the ‘gift card' as transactional. Your community research partners will offer valuable insights to create compensation strategies that simultaneously build trust and value the time of participants.

From a practical perspective, planning appropriate timelines for CER partnership activities can be challenging, as each project may vary due to budget and scope. Where possible and applicable, we recommend that timelines be co-developed with community research partners and rightsized accordingly. This requires researchers to create a space for partnership while honoring the time and capacity of community partners in critical phases of conceptualization, design, data collection, sensemaking, and dissemination. We explore this further in our next critical concept of *co-design*.

### Co-design

Building on the concepts of *trust* and *time* outlined above, *co-design* restores power and decision-making to communities that have been historically underrepresented in research, or who may perceive research as exploitive. Collaborative, co-design approaches empower communities to safely explore root causes, solutions, and share visions of the future (3) to improve systems and delivery of person-centered oral health care (26). Co-design is crucial to the success of CER.

From our experiences with CER on this project and seeing the eagerness of community to be seen, heard, and valued, we recommend integrating co-design in all stages of research. We conducted a kick-off meeting with CBOs to align our approach to this project and offered training on focus group facilitation. We feel their input on all stages of the project—from kick-off to dissemination—brought value to our project and understanding of oral health in Texas. In some cases, researchers may be critical of including partners in sensemaking (27, 28) and choose not to include community voice in soliciting feedback or interpretations. This exclusion may leave partners feeling exploited and less willing to participate in subsequent activities led by academic-focused individuals or organizations. By providing a venue and compensation for community partners to provide input during all stages, researchers demonstrate they are open to critique and recommendations—research quality and translation of findings into action.

For our project, community research partners had autonomy of their focus groups—location (virtual or in-person), date and time, method of participant compensation, and language—to create a safe and welcoming space that best meet the needs of their communities. As researchers, this may present an initial sense of discomfort due to the uncertainty that comes with sharing power typically only reserved for the ‘academic' in the partnership, and it may create challenges in navigating requirements set forth by an Institutional Review Board (IRB). This sense of discomfort and uncertainty underscores the importance of sharing best practices and strategies to encourage utilization of CER as a pathway to advancing oral health equity, ensure research integrity during co-design processes that inherently restore power to historically underrepresented communities, and address emerging concerns such as the rapidly evolving use of Artificial Intelligence (AI).

To prepare for a robust sensemaking with CBOs to contextualize findings and discuss study limitations, we conducted an initial inductive coding of qualitative data and organized emerging themes into a brief presentation. During the virtual sensemaking, we facilitated interactive discussions to explore the findings, limitations, considerations for reporting to ‘traditional' audiences (e.g., academic, public health professionals), and most importantly, returning the findings to community audiences to demonstrate how their participation is making an impact.

## Discussion

Community-engaged research (CER) provides a vital pathway to understand and address community, system, and policy barriers to the delivery of person-centered oral health care. Through active collaboration with the communities deeply impacted by barriers to timely, person-centered oral health care, CER serves a dual role—as a methodology and mechanism—that builds shared understanding, trust, and capacity essential for sustainable, impactful change. This article provides perspectives on concepts of *trust, time*, and *co-design* to guide genuine community engagement throughout the research process. A similar endeavor at the national level, “Barriers to Oral Health Care” led by the U.S Centers for Medicare and Medicaid, provides similar evidence and support for collaboration with community to advance the delivery of holistic, person-centered oral health care (29). To that end, while the alignment between these projects demonstrates the importance for continued community-engagement, more research on the practice of CER is needed. Moving forward, investments in training and capacity-building to implement CER are essential. Researchers and practitioners must be equipped with the tools and skills needed to engage communities effectively, including training in cultural humility, trauma-informed practices, and equitable partnership development. Funding mechanisms should also prioritize longer-term projects that allow for sustained trust-building, iterative collaboration, and support equitable compensation that respectfully honors the lived experience and expertise that community members bring to these efforts. Lastly, scientific venues and journals must expand their focus to highlight and share CER work, including lessons learned and best practices. Providing platforms for these efforts not only validates the importance of CER but also facilitates knowledge exchange, fosters innovation, and supports the replication and adaptation of successful strategies across different contexts.

As a result of our collaborative, community-engaged approach, we achieved a deeper understanding of the systems and conditions impacting oral health in Texas and identified opportunities within and beyond the context of clinical care to improve oral health. Despite triangulating our data sources (publicly available quantitative data, community-led focus groups, and qualitative interviews with key informants), limitations of CER include, but are not limited to the following: bias due to convenience sampling, community participants may have higher level of awareness of health resources or issues due to well-established relationships with CBOs, and the generalizability of focus groups may not be applicable for all in Texas and beyond. However, because of the input of our community research partners, we also have a deeper understanding of the study limitations.

We offer our perspectives and practical examples of CER to promote its use in public health. As such, we are thankful for our partnership with community and their trust as we continue to respectfully lift their voices in the pursuit of oral health equity for all.

## Data Availability

The original contributions presented in the study are included in the article/supplementary material, further inquiries can be directed to the corresponding author.
